# Supplementary material for: Patient Perspectives on the Care in a Long COVID Outpatient Clinic—A Regional Qualitative Analysis from Germany
Source: Healthcare (Basel). 2025 Apr 3;13(7):818. doi: 10.3390/healthcare13070818 (PMC11988876; doi:10.3390/healthcare13070818)
Supplement: Supplementary file 1 [file healthcare-13-00818-s001.zip › Table S2 Theme definition and anchor citations_v2.pdf]

Table S2: Theme definition and anchor citations

**Pathway to the specialized long COVID outpa-tient clinic (sOC)**			**Describes the paths patients took before their appointment at the sOC and how they experienced them.**	
	Care outside the sOC		Includes experiences with long COVID (LC)-related care outside the sOC with regard to the skills of the health care providers	
		Access to health care providers	Describes the pathways from patients to health care providers, the factors that shape them and perceived consequences for health status and LC-related care.	"[...] my family doctor thought I might have some-thing wrong with my lungs and also gave me a re-ferral to a lung specialist. I made a lot of phone calls. In March and got the appointments for Octo-ber, November and December. I was also very, very, very disappointed." (I05, line 177 ff.)
		care experienced as inadequate	Describes LC-related care outside the sOC that patients perceive as inadequate and perceived consequences for health status and LC-related care	"Because even the GP, it was new for her, she didn't really know what the next steps were, who I could turn to, who she could turn to, it was all still a bit uncertain." (I09, lines 15 ff.)
		Openness to LC	Describes patients' perceived willingness of health care providers to participate in LC care and perceived consequences for health status and LC-related care	"And the [GP] said: "No. You have Long COVID. I've now read the report, I've also read up a bit on what it is. [...]" And then he put me on sick leave until the end of the month." (I05, lines 130 ff.)
	Own initiative and personal responsi-bility		Describes that patients themselves are actively involved in their LC-related care with the aim of improving their health status and LC-re-lated care.	"So as far as I could with the limited strength, I just did some research >laughs< on the Internet and then - and yes, I downloaded things from the Char-ité website [...] I then started pacing on my own based on the recommendation from the Charité." (I03, lines 25 ff.)
	Appraisal of the stepped care ap-proach		Describes how patients assess the expert rec-ommendation that GPs should be the first point of contact for LC, provide primary care and, if necessary, involve other treatment	"Yes, I think it would be good, because it makes sense, because it's also about access and you would also have access.<br>access. I don't think they can implement it in terms

			partners such as specialists, therapists or sOC in the care.	of capacity, the GPs, at least from my feeling. " (I06, line 156 f.)
**Care provided in the sOC**			describes LC medical care within the sOC from the perspective of LC patients	
	Expectations of the appointment in the sOC		describes the assumptions under which patients attended their appointment at the sOC and the extent to which these were fulfilled.	"[...] so on the one hand I tried to have few expectations, but I had high ones, because I knew it was Heidelberg, and I had, above all I hoped that, simply, my only goal was to talk to someone who knew about it, who was interested in it, who was researching it, who had undergone further training in these areas, simply to be able to talk to experts about recommendations and - as a higher authority, simply, compared to the doctors in private practice." (I06, line 177 ff.)
	Process of the appointment at theOC		Describes how patients experienced the process and duration of the appointment at the sOC.	
		Waiting time for the appointment at the sOC	records statements about how much time has passed between contacting the sOC and the appointment at the sOC.	"And then I called the Long COVID outpatient clinic and they said straight away: "No more appointments. We're full. We're not taking anyone." And then of course I was a bit depressed [...]" (I02, line 40 f.)
		Course and duration of the appointment at the sOC	Describes how patients experienced the process and duration of the appointment at the sOC.	"[...] I was told right at the beginning where I had to go and I couldn't remember it at all. I then had to ask again where I had to go, exactly where the blood test was, and then back down again. And then, in the meantime, where you always had to wait, I had time to fill out the questionnaires, and then I had to wait again [...]" (I08, lines 79ff.)
	Experienced competences		Describes how patients perceived and assessed the LC-related knowledge and methods of sOC staff and their application, as well as their social skills.	"We have the concentrated expertise in the Long COVID Outpatient Clinic, we have people there who go to relevant conferences in international contexts during the year, where everyone is discussing certain topics and so on. The doctors complete this, come here and are then in his Long COVID outpatient clinic and can pass this on

				accordingly. And pass it on very, very well." (I07, lines 348 ff.)
	Differences to LC care outside of the sOC		Includes explicit statements from patients about the extent to which care in the sOC differed from care outside the sOC	"Well, there was just this systematic approach. And you simply noticed that I wasn't the first patient to show up with the full range of symptoms" (I12, line 211 f.)
	Consequences of the appointment at the sOC		Describes the importance of the conception in sOC with regard to the health status of patients and their future LC-related care	
		Direct health consequences	describes the immediate consequences that patients attribute to the appointment at the sOC for the patient's state of health.	"For me, that was actually >breathe out loud<, so the whole day was a complete crossing of boundaries for me. So, the most massive one I've ever had." (I11, line 107 f.)
		Consequences for further care and acceptance of the disease	describes the prospective consequences that patients attribute to the appointment at the sOC for their health and their future medical LC care.	"And that really, really helped me. Also this, this impetus to do this KG on the device now." (I10, line 180 f.)
	single appointment at the sOC			
		Evaluation of onenetime appointment at the sOC	Describes how patients assess the current regulation that they only receive one appointment at the sOC.	"What's not so great, of course, is that you only get one appointment because there isn't enough capacity. So you can't somehow say: "I'd like to come back in three months and discuss it further then." (I03, Z.95 ff.)

		Possible reappointment at the sOC	Describes the reasons why and when patients would decide to return to the sOC.	"I'll say now, if I don't feel any big improvements physically by the end of quarter one, then I'd just get in touch again and say: "Hey, maybe we need to start somewhere else."" (I10, line 205 ff.)
	Indications for an appointmentat the sOC		describes at what times and for what reasons patients would advise other LC patients to visit the sOC.	"When you realize yourself that you're not getting anywhere, that your GP might say: "I don't know. There's nothing. You'll have to wait until you feel better" or "You'll have to sit it out." That you then try to get an appointment at the long-COVID out-patient clinic. And, as I said, maybe get another tip or other help there." (I01, line 229 ff.)

sOC: specialized long COVID outpatient clinic; LC: long COVID
