# Supplementary material for: Patient Perspectives on the Care in a Long COVID Outpatient Clinic—A Regional Qualitative Analysis from Germany
Source: Healthcare (Basel). 2025 Apr 3;13(7):818. doi: 10.3390/healthcare13070818 (PMC11988876; doi:10.3390/healthcare13070818)
Supplement: Supplementary file 1 [file healthcare-13-00818-s001.zip › Table S1 Interview guide_v3.pdf]

Table S1: Interview guide

Topic	Question
Transition	*Let's start with a look back at your history with Long COVID.*
Determination long COVID	**How did you come to be diagnosed with long COVID**?<br>• What exactly was done?<br>• Who was involved?<br>**What was done after you were diagnosed with long COVID?**
Access to the long COVID specialized outpatient clinic	**How did it come about that you attended an appointment at the long COVID specialized outpatient clinic?**<br>• Who came up with the idea? When?<br>• How did the appointment process work?<br>• How did the coordination between the general practice and the outpatient clinic work?<br>• What expectations were associated with the appointment in the outpatient clinic?
Transition	*Thank you very much for this report on your story. I would also like to know more about how you found the outpatient clinic.*
Experience with and in long COVID specialized outpatient clinic	**How did you experience your visit to the long COVID specialized outpatient clinic?**<br>• How did you experience the processes?<br>• To what extent did you notice differences compared to your treatment of Long COVID beforehand? (examinations, recommendations, discussions, organizational matters)<br>**How were you able to implement the recommendations of the outpatient clinic in the time after the appointment** / How were you able to initiate xy?<br>• What helped you with this?<br>• What was difficult for you?<br>**When you think back to the appointment, what effect did the visit to the outpatient clinic have on you?**<br>• How has your knowledge of the disease perhaps changed as a result of the appointment at the outpatient clinic?<br>• To what extent has your view of long COVID changed?<br>**So to what extent have your expectations of the outpatient clinic been met?**<br>**What situations can you imagine in which it would make sense for you to return to the long COVID specialized outpatient clinic?**<br>• At what interval would you personally find it useful to introduce yourself again?<br>**What would you recommend to other patients? In your opinion, when should people attend an appointment at the outpatient clinic?**
Transition	*I have now heard a lot about your personal experience and would like to think a bit bigger and talk to you about long COVID care in a more general way. You may already know that the Rhine-Neckar long COVID Network exists. This also includes the website longcovidnetz.de You may have heard of it before. Various doctors and therapists have joined forces in the network so that they can work together here in the region to provide care for Long COVID patients.*
Long-COVID Network Rhine-Neckar	**What do you think when you hear about this network?**<br>• What do you already know about the network?<br>• How did you first hear about the network?<br>• To what extent would you say you have used the network?<br>• To what extent did the network support you?
Transition	*The network also wants to better structure the paths that Long COVID sufferers take in our healthcare system and implement what many experts recommend: namely that GPs are the first point of contact for Long COVID and provide primary care. (If there is a further need, GPs can also involve other doctors or therapists at various levels).*
Stepped care approach	**What do you think of the fact that this is recommended, i.e. that general practitioners should be the first point of contact?**<br>• What do you think is good about it?<br>• What do you think is not so good about it?
Final question	**That was all the questions I had. What else do you think is important about the topics we have discussed that has not yet been mentioned?**<br>*Thank you very much for taking part in this interview. You have given me a lot of insight into your experiences with care at long COVID.*
